# Innovation and Collaboration in Plastic Surgery

**DOI:** 10.1055/s-0043-1778669

**Published:** 2024-01-29

**Authors:** Peter C. Neligan

**Affiliations:** 1 Division of Plastic Surgery, University of Washington, Seattle, Washington

**Figure:**
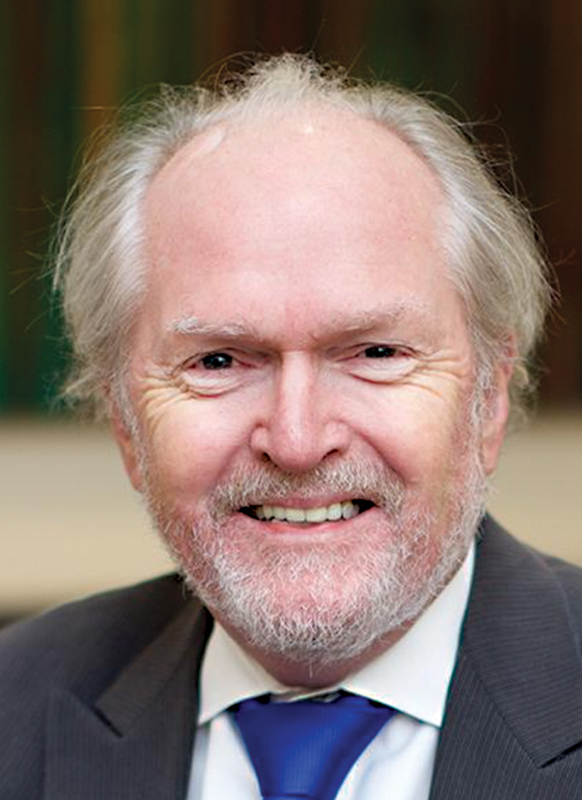
Peter C. Neligan

Collaboration (from Latin *com-* “with” + *laborare* “to labor,” “to work”) is the process of two or more people, entities, or organizations working together to complete a task or achieve a goal. 1 Collaboration in surgery, while it happens, has not traditionally been the norm because most specialties do not need to work together. However, because of the nature of Plastic Surgery, interaction with other specialties is the norm. But, collaboration goes beyond mere interaction. It means taking part in the management of each case, attending tumor board/planning meetings, and being part of the team, not just showing up in the operating room at the end of a case. The concept of collaboration in surgery was brought home to me many years ago. I was attending Head and Neck tumor board rounds. A neurosurgeon presented a Skull Base case and concluded that the patient's tumor was likely resectable but access was, in his estimation, fraught with hazard. The head of Otolaryngology, Head and Neck Surgery said, “I can get you in there but it'll leave a hell of a hole.” I then suggested “I can close the hole.” We looked at each other and realized that together we could achieve what neither one of us could do alone. The case went well and the three of us regularly collaborated for many years to come. We introduced the use of free tissue transfer to Skull Base surgery and consequently reduced the incidence of the three biggest complications of that surgery; brain abscess, meningitis, and CSF leak. 2 However, with the development of endoscopic techniques, skull base defects were being created with minimal access and the difficulty of introducing a free flap into these defects led to a resurgence of the very complications that free tissue transfer had mitigated; brain abscess, meningitis, and CSF leak! This led to the development of alternative local and regional flaps that could be used to close endoscopically created skull base defects. 3 4 An innovation in surgical technique led to an innovation in flap development; another lesson for me. But more of innovation later.

So why there is not more collaboration in surgery? There are several reasons. One reason is money. When I moved my practice from the University of Toronto to the University of Washington in Seattle in 2007, I wanted to continue to be involved in head and neck reconstruction which made up approximately 70% of my practice in Toronto. In Seattle, the Chief of the Head and Neck Service, who is a friend, told me ruefully that if he sought my involvement in his reconstructions, the consequent revenue would be lost to his department. So, he continued to do his own reconstructions. That made me realize that getting another surgical department involved in patient management may reduce revenue for the original department. The silo organization of the various departments in this case tended to stifle collaboration for economic reasons. There is, of course, no reason why Head and Neck surgeons should not do their own reconstructions. Neither is there any reason for any specialty not to complete all aspects of a given case but, as I have discovered in my career, sharing expertise is frequently more beneficial for the patient and, indeed, for the discipline of surgery as a whole.

Marketing a team approach may mitigate individual financial downsides. An example of this is the Mayo Clinic model wherein patients go to the institution rather than to an individual physician in the Mayo Clinic. The Clinic has the name recognition and reputation, rather than the individual physician.

Another reason why collaboration may not be more ubiquitous is that of availability. To give a Plastic Surgery example, let us consider the case of postmastectomy breast reconstruction. A breast surgeon who has done a mastectomy waits for the plastic surgeon, who may be busy in another operating room, to come and place a tissue expander for the first stage of breast reconstruction. (S)he thinks, “why don't I put it in myself? It can't be that hard.” It is not! That is the thin end of the wedge that may ultimately lead to the plastic surgeon no longer being asked to do the reconstruction. The next time the breast surgeon will insert the tissue expander without consulting the plastic surgeon and will probably go on to replace the tissue expander with a permanent implant eventually. While (s)he is certainly perfectly capable of doing this, (s)he is depriving the patient and him/herself of expertise that is likely going to result in a better outcome. This circumstance can prevail whenever a procedure calls for the involvement of several services. Because of the complexity of scheduling, it is much easier to schedule a case that involves just one service. It may be easier but is it better?

The final barrier to collaboration is ego. How do you know you have an ego problem? Nouman Ali Khan is a Pakistani–American Islamic speaker and Arabic instructor who founded the Bayyinah Institute for Arabic and Qur'anic Studies after serving as an instructor of Arabic at Nassau Community College. He has been named one of the 500 most influential Muslims in the world by the Royal Islamic Strategic Studies Centre of Jordan. 5 He said, “If someone corrects you, and you feel offended, then you have an ego problem.” My own definition of an ego problem is “if your e-mail signature is longer than your e-mail. You have an ego problem!” In a collaborative team there is no room for ego and the members of such a team need to recognize and respect the abilities of each of the other members of the team.

Consider innovation again. According to Wikipedia, *Innovation* is the practical implementation of ideas that result in the introduction of new goods or services or improvement in offering goods or services. 6 Surgeons innovate all the time. Most of the time this innovation is incremental. We change the way we put a suture in, we modify an incision, we design a flap differently. We do this to tweak what we do and improve our outcomes and we adopt those innovations into our practice. Sometimes we innovate to deal with a specific problem that we have not been faced with before. Innovation occurs most frequently in times of war and natural disaster when we are faced with patients and situations in such numbers that we have to find new methods of treatment, through innovation, just to cope. What all these innovations have in common is the desire to improve things or to treat conditions that we were hitherto unable to tackle. This is something we all want. While the majority of innovations are incremental, some are more radical, completely changing an approach, often developing new techniques that seem to break the rules we have lived by up until that point. One example is the work of Paul Tessier. His innovation is best described as radical. He broke all the rules of surgical dogma by combining an intracranial and extracranial approach for the treatment of hypertelorbitism. 7 This innovation led to the development of the subspecialty of Craniofacial Surgery and fostered the advancement of the related specialties of neurosurgery, maxillofacial, and ophthalmic surgery. I have mentioned that developments in one specialty can lead to advances in another. A great example of this is the application of bone lengthening to the craniofacial skeleton. The possibility of lengthening bone was discovered by Gavriil Ilizarov in 1951. 8 This proved a revolutionary advance. Joseph McCarthy subsequently adapted that technique to the craniofacial skeleton and distraction osteogenesis, as it is now called, has changed the way we treat many craniofacial anomalies. 9 In the history of Plastic Surgery, there have been several major innovations. Innovation is not confined to reconstructive surgery. Dr. Sydney Coleman introduced the concept of structural fat grafting in aesthetic surgery. 10 This was such an innovative advance that fat grafting is now widely practiced in both aesthetic and reconstructive surgery. An unforeseen benefit of fat grafting has been recognized in its mitigation of radiation fibrosis, particularly in the breast. 11

Innovation has been responsible for many of the great advances in surgery and while research has, of course, played a key role in development, innovation is at least as important. While we like to think that research comes first and spawns innovation, in practice it is often the other way round. We do something new and then go to the laboratory to figure out how it works. Taylor laid the ground work in describing the angiosomes of the human body in a series of elegant lead oxide cadaver studies. 12 That opened the door for the development of perforator flaps in reconstructive surgery. 13 Sometimes one would raise a flap on a given perforator (the innovation) and then go to the laboratory to figure out what other vessels were available and how much these perforators would perfuse (the research). 14 15

As I have described, innovation and collaboration are often linked. There are several prerequisites to making collaboration successful. I like to refer to them as the four As: Availability, Affability, Acknowledgment, and Ability. Availability has already been discussed as one of the potential barriers to successful collaboration. Affability is extremely important. Nobody wants to work with a grouch. Working together should be a pleasant experience. Acknowledgment can also be described as give and take; accepting the value of each member's contribution. Ability is also obvious. One has to bring added value to the team and that value is ability and expertise.

Collaboration is sometimes referred to as teamwork. I once had to give a talk on teamwork which I titled “There is no 'I' in TEAM.” The concept of a collaborative team is often misrepresented. I have heard surgeons say, “I have a great team” when what they really mean is “I have all these people working for me who do things exactly as I want them.” Now while there is a place for such a team to improve efficiency, the real concept of a team is to accomplish together what no one individual on the team could do alone and there is evidence to show that multidisciplinary teams make fewer errors, are more patient-centered, and more efficient than monodisciplinary teams. 16 17 The exchange of ideas and techniques fosters innovation and advance. Playing in a collaborative team sometimes means compromise, standing back, and letting someone else do something that you think you could do yourself. As surgeons, we are not very good at that. However, as I have experienced over my career, it pays off hugely.
